# Bumblebees Express Consistent, but Flexible, Speed-Accuracy Tactics Under Different Levels of Predation Threat

**DOI:** 10.3389/fpsyg.2018.01601

**Published:** 2018-09-03

**Authors:** Mu-Yun Wang, Lars Chittka, Thomas C. Ings

**Affiliations:** ^1^Department of Biological and Experimental Psychology, School of Biological and Chemical Sciences, Queen Mary University of London, London, United Kingdom; ^2^Graduate School of Arts and Sciences, The University of Tokyo, Tokyo, Japan; ^3^Institute for Advanced Study, Berlin, Germany; ^4^Department of Biology, Anglia Ruskin University, Cambridge, United Kingdom

**Keywords:** animal personality, *Bombus terrestris*, predation risk, predator crypsis, speed-accuracy trade-offs

## Abstract

A speed-accuracy trade-off (SAT) in behavioural decisions is known to occur in a wide range of vertebrate and invertebrate taxa. Accurate decisions often take longer for a given condition, while fast decisions can be inaccurate in some tasks. Speed-accuracy tactics are known to vary consistently among individuals, and show a degree of flexibility during colour discrimination tasks in bees. Such individual flexibility in speed-accuracy tactics is likely to be advantageous for animals exposed to fluctuating environments, such as changes in predation threat. We therefore test whether individual speed-accuracy tactics are fixed or flexible under different levels of predation threat in a model invertebrate, the bumblebee *Bombus terrestris*. The flexibility of speed-accuracy tactics in a foraging context was tested in the laboratory using a “meadow” of artificial flowers harbouring “robotic” crab spider predators. We found that while the ranking of bees along the speed and accuracy continuums was consistent across two levels of predation threat, there was some flexibility in the tactics used by individual bees – most bees became less accurate at colour discrimination when exposed to predation threat when flower types were rewarding. The relationship between decision speed and accuracy was influenced by predator detectability and the risk associated with making incorrect choices during the colour discrimination task. Predator crypsis resulted in a breakdown in the relationship between speed and accuracy, especially when making an incorrect floral choice incurred a distasteful quinine punishment. No single speed-accuracy tactic was found to be optimal in terms of foraging efficiency under either predation threat situation. However, bees that made faster decisions achieved higher nectar collection rates in predator free situations, while accurate bees achieved higher foraging rates under predation threat. Our findings show that while individual bees remain relatively consistent in terms of whether they place greater emphasis on speed or accuracy under predation threat, they can respond flexibly to the additional time costs of detecting predators.

## Introduction

Choices made by animals frequently involve a trade-off between decision speed and decision accuracy (Wickelgren, 1977; Chittka et al., 2009; Heitz and Schall, 2012), with fast decisions tending to be less accurate than slow decisions for a given task condition. A speed-accuracy trade-off (SAT) has been shown to occur during discrimination tasks across a wide range of taxa including humans (Simen et al., 2009; Bogacz et al., 2010), non-human primates (Heitz and Schall, 2012), birds (Ducatez et al., 2015), fish (Wang et al., 2015), and insects such as bees (Chittka et al., 2003; Burns and Dyer, 2008). The majority of studies on SAT, especially in humans and non-human primates, have used the SAT as a paradigm for exploring behavioural flexibility in decision making and choice behaviour (e.g., Fitts, 1966; Wickelgren, 1977) and its neuronal basis (reviewed by Standage et al., 2014 and also see Heitz and Schall, 2012; Hanks et al., 2014 for non-human primate examples). More recently, researchers working on animals, including bees, birds, and fish (Chittka et al., 2003; Ducatez et al., 2015; Wang et al., 2015; but also see Phillips and Rabbit, 1995 for a human example), have also considered SAT from a different perspective, i.e., whether the SAT is a stable trait in which the individual differences are maintained within a population over time.

Response speed has long been an important component describing individually consistent behavioural traits in vertebrates, such as shyness-boldness or neophobia (Van Oers et al., 2005; Toms et al., 2010). These traits, related to response speed, can be heritable (Drent et al., 2003) and different traits can be adaptive depending upon environmental changes (Dingemanse et al., 2004). In invertebrates, this approach has recently been used to consider the relationship between decision speed and accuracy. For example, Chittka et al. (2003) showed that foraging bumblebees express inter-individual variation in speed-accuracy tactics during floral colour discrimination. This variation remained consistent even when the cost of making errors increased, although all bees became slower and more accurate. Similar individual variation in speed-accuracy tactics has also been shown in honeybees (Burns and Dyer, 2008). While these studies indicate that speed-accuracy tactics in invertebrates do vary consistently among individuals, and that there is some flexibility at the level of the individual (Chittka et al., 2003), we still have limited understanding of how they can be adjusted to match changing situations (Chittka et al., 2009), such as increased predation threat.

Levels of inter-individual variability of behavioural phenotypes (O’Steen et al., 2002; Dingemanse et al., 2004) and individual behavioural flexibility (Herborn et al., 2014) are believed to be influenced by environmental fluctuation. We would therefore expect selection to favour flexibility in speed-accuracy tactics in social animals, such as bumblebees, adapted to dynamic environments (reviewed in Klein et al., 2017) where factors such as food availability and predation risk vary temporally and spatially. Bumblebees are social insects where the worker caste collects food (nectar and pollen from flowering plants) for the entire colony (Goulson, 2003). Foraging bees maximise their foraging efficiency by processing visual and olfactory cues to select the flowers of plants that provide the greatest returns (Chittka et al., 1999; Chittka and Raine, 2006). However, the best available options vary considerably through time and space (Von Buttel-Reepen, 1900; Arnold et al., 2009), and foraging bees need to avoid predators such as the crab spider *Misumena vatia* which hunts on flowers (Morse, 2007). The risk of predation from these predators also varies from patch to patch (Morse, 2007) and, due to their ability to change colour, the detectability of the spider can vary depending upon the colour of the flower it is hunting on (Chittka, 2001).

We therefore use a model invertebrate, the bumblebee *Bombus terrestris*, and an established predator avoidance learning paradigm (Ings and Chittka, 2008, 2009), to examine the flexibility of individual speed-accuracy tactics in response to changing predation risk. Bees are exposed to a natural scenario where they have to discriminate between two similar flower types to maximise energy intake in an artificial meadow where the risk of predation by model crab spiders is added half way through the experiment. In this design, introduction of predation risk changes the decision task slightly. We therefore do not focus on classical SAT, where participants perform the same task in different motivational conditions (speed or accuracy emphasis), but rather, we focus on inter-individual variation and intra-individual consistency in decision speed and accuracy under changing predation risk. Our main questions are: (1) Do bees maintain consistent speed-accuracy tactics in a floral colour discrimination task when exposed to increased predation risk? (2) Does the optimal speed-accuracy tactic change with predation risk and the difficulty of detecting predators? We hypothesise that the speed-accuracy tactic employed by individual bees will be flexible and that the optimal speed-accuracy tactic will differ depending upon predator crypsis and costs of incorrect choices in flower colour discrimination.

## Materials and Methods

### Study Animals

Bumblebees (*B. terrestris dalmatinus*, Dalla Torre 1882) from three colonies obtained from a commercial supplier (Syngenta Bioline Bees, Weert, Netherlands) were used in the experiment. Individual bees were marked with numbered tags (Christian Graze KG, Weinstadt-Endersbach, Germany). All colonies were maintained at room temperature (23°C) and exposed to a 12:12 h light/dark cycle, with the light phase starting at 8 am. All colonies were supplied with *ad libitum* sucrose solution (50%, v/v) and pollen.

### Experimental Apparatus

**FIGURE 1:**
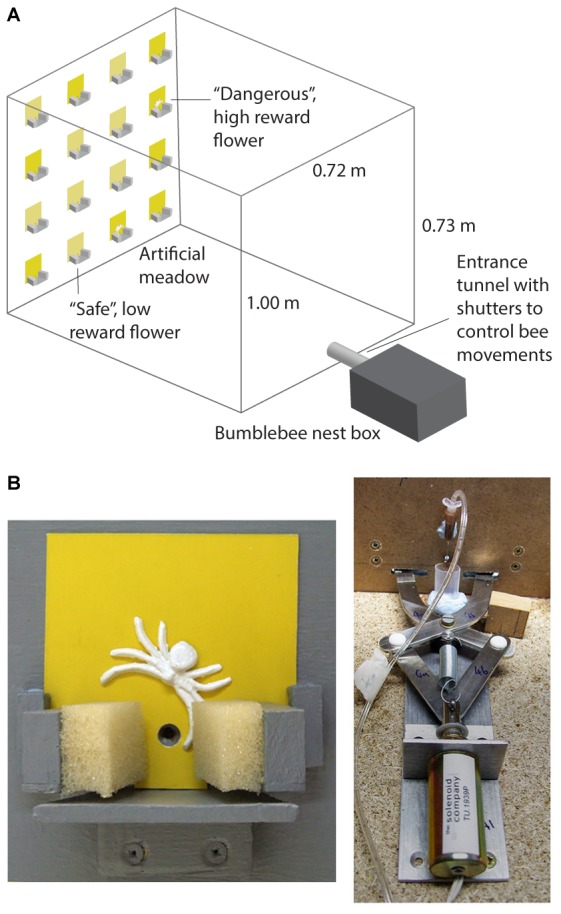
The predator avoidance paradigm used in the experiments. **(A)** Shows the artificial meadow in the flight arena for bees in the conspicuous spider group. **(B)** Shows a close-up photograph of a dangerous flower depicting the floral display (7 cm × 7 cm) with a conspicuous 3D spider model and sponge coated pincers (left panel). The panel to the right shows the solenoid operated trapping mechanism behind the flower (this closed the sponge coated pincers for 2 s when a bee lands to feed). It also shows the syringe tip which dispenses the sucrose or quinine solutions.

Full details of the experimental apparatus are provided in Ings and Chittka (2008) and Ings and Chittka (2009). This experiment was carried out in a wooden flight arena (l = 1 m, *w* = 0.72 m, and *h* = 0.73 m) with a UV-transmitting Plexiglas lid. Controlled lighting was provided by two twin lamps [TMS 24 F with HF-B 236 TLD (4.3 kHz) ballasts, Philips, Netherlands], fitted with Activa daylight fluorescent tubes (Osram, Germany), which were suspended above the flight arena. A four by four vertical array of artificial flowers (7 cm × 7 cm flat cards painted with yellow acrylic colours) was presented on a grey background on the end wall of the arena (**Figure 1A**). Bees entered the arena through an entrance tunnel attached to the opposite wall to the meadow. Each artificial flower (**Figure 1B**) consisted of a small wooden landing platform (40 mm × 60 mm), 10 mm under a small hole through which bees could access rewards (sucrose solution). Syringe pumps (KD Scientific, KD200, Holliston, MA, United States) were used to provide a continuous supply of sucrose solution at the tips of 26G syringe needles (BD Microlance Drogheda, Ireland; 0.45 mm × 13 mm) placed behind the access hole on the flowers.

To simulate predation risk, robotic “spider arms” (custom-built by Liversidge & Atkinson, Romford, United Kingdom) covered with sponges (**Figures 1A,B**) were set up at the base of the flowers to simulate predation attempts (detailed in Ings and Chittka, 2008, 2009). To provide realistic visual predator cues, a 12 mm wide three dimensional model (made from Gedeo Crystal resin) of a crab spider (*M. vatia*) was placed just above the feeding hole on the “dangerous flowers” (**Figure 1B**). Full details of the dangerous flowers, including spectral reflectance of the background, spiders and flowers can be found in Ings and Chittka (2008) and Wang et al. (2013).

### Pre-training

To allow bees to become accustomed to the arena and flowers, all bees were given unrestricted access to the flight arena for a minimum of 1 day prior to the beginning of the experiments. No floral signals were placed in the artificial meadow to avoid bees developing any colour bias prior to the experiments. However, all flowers were supplied with a constant flow (1.85 ± 0.3 μl per minute) of 50% (v/v) sucrose. Individual bees that had continued feeding for a minimum of three foraging bouts (i.e., they entered the arena, collected sucrose solution from the artificial flowers and returned to the nest on at least three consecutive occasions) were used in the experiments.

### Experimental Design

**FIGURE 2:**
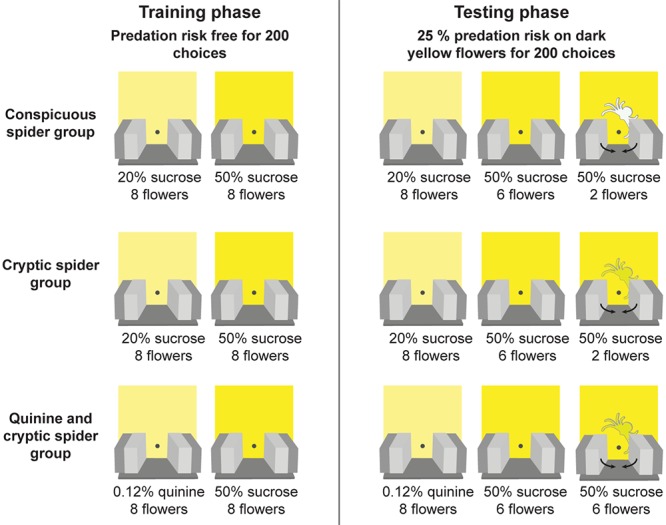
Protocol for the experimental design. Three groups of bees were initially trained (training phase) to discriminate between two similar shades of yellow artificial flowers in a predator free environment. In the first two groups (“conspicuous spider” and “cryptic spider”) the two flower types contained different quality of sucrose reward (20% v/v in the light yellow versus 50% v/v in the dark yellow). In the third group (“quinine and cryptic spider – hereafter referred to as just ‘quinine’”), the dark yellow flowers contained a sucrose reward (50% v/v sucrose) and the light yellow flowers contained a bitter quinine punishment [0.12% (w/v) quinine hemisulfate solution]. After bees made 200 choices during training they entered the testing phase where they were exposed to predation risk from model crab spiders, which were present on 25% of the dark yellow (high reward) flowers. Spider models were either easily detectable (the conspicuous spider group) or difficult to detect (the cryptic spider and quinine groups). If a bee visited a flower with a model spider it was captured for 2 s by the robotic arms.

Full details of the experimental procedure are provided in Wang et al. (2013) and summaried in **Figure 2**. During the training phase, the 7 cm × 7 cm floral signals were added to the artificial meadow (**Figure 1A**). Bees were then trained to distinguish between two similar shades of yellow artificial flowers (for details of the colours see Wang et al., 2013) for 200 flower choices - a bee needed to land on the platform of a flower and probe for artificial nectar to be deemed a choice. In two groups (conspicuous spider and cryptic spider), the dark yellow flowers were more rewarding [50% (v/v) sucrose solution] than the light yellow flowers [20% (v/v) sucrose solution]. To encourage flower discrimination in a third group (quinine and cryptic spider – hereafter referred to as just ‘quinine’), we replaced the 20% (v/v) sucrose solution with a distasteful 0.12% (w/v) quinine hemisulfate solution that bees are known to rapidly learn to avoid (Chittka et al., 2003). In the testing phase, we introduced spider models, either highly conspicuous (white; conspicuous spider group) or cryptic (same colour as the flowers; cryptic spider and quinine groups) to two of the eight (i.e., 25%) high quality (dark yellow) flowers and tested the bees for another 200 choices. Three to six foraging bouts were required for bees to make 200 choices, and bees were allowed to complete their final foraging bout and return to the nest (see Wang et al., 2013 for further details). When bees landed on “dangerous” flowers (with a spider model), they were immediately exposed to a simulated predation attempt by being held by the arms of a “robotic crab spider” for 2 s – thus they had no opportunity to collect the sucrose solution. The positions of the flowers were changed in a pseudo-random fashion between each foraging bout (at least three were required to attain 200 choices): positions of different flower types were changed randomly, but the number of high reward flowers in both the top and bottom two rows of the meadow were maintained the same in order to avoid spatial preference bias.

### Data Analysis

The movements and positions of 44 bees from across the three colonies were recorded in real time during the experiment. Of these, four were excluded from the analyses as they stopped foraging during the experiment (this left 15 bees in each of the conspicuous spider and cryptic spider groups and 10 bees in the quinine group). Three-dimensional coordinates of bee positions were calculated 50 times per second using two video cameras connected to a computer running Trackit 3D software (BIOBSERVE GmbH, Bonn, Germany). We calculated the time bees spent in the investigation zones, which were 7 cm (length) by 9 cm (width) by 9 cm (height) from the holes providing sucrose or quinine solution. Investigation zones were set based on the visual angles of bumblebees where bees were able to detect both flower signals and predators using colour contrast (Spaethe et al., 2001).

To remove learning effects, we calculated the colour discrimination accuracy (proportion of high reward flowers chosen) and decision speed during the final 30 choices (out of 200) made during each phase of the experiment. In the testing phase, only visits to high reward flowers without spiders were scored as correct choices. We used the average time spent inspecting flowers (duration in the investigation zones), rather than average time between choices (e.g., as in Chittka et al., 2003), as our measure of decision speed. Time between flowers is only a proxy of decision time and is influenced by other factors such as flight speed and path length between flowers. Prior to analysis, inspection time was converted to relative decision speed using the following formula: Speed = 1 – [(decision time – minimum decision time)/(maximum – minimum decision time)]. Thus the bee that took the longest (1.31 s per flower) to inspect flowers was scored as 0 and the fastest bee (0.31 s per flower) was scored as 1 (mean ± 1SE decision time = 0.60 ± 0.2 s per flower). All statistical analyses described below were carried out in RStudio 1.1.423 (R Core Team, 2015) running R 3.4.3 (R Core Team, 2017).

### The Relationship Between Speed and Accuracy

Before examining the consistency of floral colour discrimination speed-accuracy tactics across situations (change in predation risk), we used linear correlation analysis (Pearson’s product moment correlation) to determine if speed and accuracy were related. Correlation was used as there was no *a priori* reason to expect decision speed to be dependent upon accuracy or *vice versa*. Each experimental group was examined separately. Normality tests (Shapiro–Wilk) and visual inspection of the scatterplots were undertaken to check that the assumptions of linear correlation were not violated.

### Consistency of Speed-Accuracy Tactics Under Different Levels of Predation Risk

To test whether floral colour discrimination speed-accuracy tactics are rigid or flexible with changing predation risk, we used a combination of linear regression and paired *t*-tests. While both speed and accuracy are proportions, inspection of the residuals from fitted models, along with normality tests (Shapiro–Wilk tests) showed that it was not necessary to transform these data or use generalised linear models. Linear regression was used to identify whether speed and accuracy during the testing phase were dependent upon speed and accuracy during the training phase (each group was analysed separately). The associated *R*^2^-values provide an index of the stability of the rank position of each bee in terms of speed or accuracy, i.e., whether the fastest bee remains the fastest bee under predation risk. The *t*-tests provide a measure of the consistency of the magnitude of speed and accuracy for individual bees within each group. Thus, if bees in a group maintained consistent speed-accuracy tactics under predation threat they would have high *R*^2^-values but low *t*-values. For the regression analysis, residual versus fitted value and quantile–quantile plots were inspected to check that each model met the assumptions of linear regression. The “linearHypothesis” function in the R package “car” version 2.14 (Fox and Weisberg, 2011) was used to test whether slope coefficients differed significantly from 1 (all bees remained equally consistent). The slope coefficient values were used to indicate effect sizes of the regressions and Hedges g was calculated in version 0.7.1 of the “effsize” package (Torchiano, 2017) in R to assess effect sizes for the mean differences in speed and accuracy between training and testing phases.

### Optimal Tactic Under Different Levels of Predation Risk

In a predation free environment, the optimal speed-accuracy tactic should yield the highest nectar collection rates. However, in an environment with a high predation risk, the optimal tactic will involve a trade-off in terms of nectar collection rate and avoiding being killed by predators. However, because all bees had a strong predator avoidance response by the end of training (Wang et al., 2013), there was insufficient variation to allow a meaningful analysis of the influence of speed-accuracy tactics on predation risk. Thus, we focused on nectar collection rates as our measure of optimality in relation to speed-accuracy tactics.

Nectar collection rate (mg sucrose per second) was calculated by dividing the amount of sucrose collected by each individual bee by the total time they spent foraging in the arena. Each of the high reward flowers provided approximately 4 mg of sucrose (4.7 μl of 50% v/v sucrose solution) while each low reward flower provided approximately 0.8 mg of sucrose (4.7 μl of 20% v/v sucrose solution). Therefore, during training there was on average 80% more sugar reward available in the high reward flowers compared to the low reward flowers. During testing, this difference reduced slightly to 73.3% as no sugar could be collected from dangerous flowers. Flowers containing quinine solution provided no sucrose reward, and when bees visited a flower harbouring a crab spider model they were captured before they could collect any sucrose solution.

First, we examined the relationship between decision time and total foraging duration using Pearson’s product moment correlation. To meet the assumption of normality, total foraging duration was log (natural) transformed prior to the analysis. The influence of decision speed and accuracy on nectar collection rate was examined using separate general linear models for training and testing phases. Full models (including interactions) with experimental group and both speed and accuracy were initially fitted to determine which variable explained the greatest amount of variation. We included experimental group in the model to test whether the intercepts or slopes differed among the experimental treatments. After fitting the full model (including the interactions between group and speed and accuracy) we used the “Anova” function in the R package “car” to calculate Type III sums of squares to allow us to choose which variables to drop to improve the fit of the models. Model terms were dropped sequentially until we arrived at the minimum adequate model with the lowest Akaike Information Criterion (AIC). Residual versus fitted value plots, quantile–quantile plots and variance inflation factors (VIF) were inspected to check for any violations of model assumptions. We used the same procedure to test whether nectar collection rate during testing was dependent upon nectar foraging rate during training. The “linearHypothesis” function in the R package “car” was used to test whether slope coefficients differed significantly from 1 (all bees remained equally consistent).

## Results

### The Relationship Between Speed and Accuracy

**FIGURE 3:**
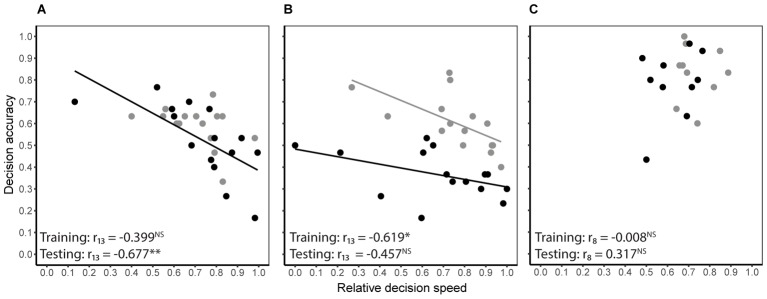
Relationship between decision speed and accuracy of individual bees for the conspicuous spider **(A)** and cryptic spider **(B)** groups where high reward flowers contained 50% sucrose and the low reward flowers contained 20% sucrose, and the quinine group **(C)**, where rewarding flowers contained 50% sucrose and the distasteful flowers contained quinine. Grey symbols represent the predation risk free training phase, and black symbols represent the testing phase where bees in the conspicuous spider group were exposed to predation threat from conspicuous spiders and bees in the cryptic spider and quinine groups were exposed to cryptic spiders. Solid lines represent significant linear fits (NB the lines are not extrapolated beyond observed values). Statistical significance of Pearson’s correlation coefficient for each series is indicated with stars (^∗^*P* ≤ 0.05, ^∗∗^*P* ≤ 0.01, NS for *P* > 0.05).

Decision speed (inverse of decision time) and accuracy were generally negatively correlated during both training and testing, i.e., some bees were slow and accurate, while others were fast and error prone. However, the degree of correlation differed between treatment groups (**Figures 3A–C**). In the absence of predation risk, speed and accuracy were significantly correlated in the cryptic spider group (Pearson’s *r*_13_ = -0.619, *P* = 0.013) but not the conspicuous spider group (Pearson’s *r*_13_ = -0.399, *P* = 0.141) or the quinine group (Pearson’s *r*_8_ = -0.008, *P* = 0.982). When the conspicuous spider and cryptic spider groups, which experienced identical conditions during training, were pooled, the overall relationship between speed and accuracy was strongly negatively correlated (Pearson’s *r*_28_ = -0.525, *P* = 0.003). Under predation risk, speed and accuracy were negatively correlated in the conspicuous spider group (Pearson’s *r*_13_ = -0.677, *P* = 0.006) but not the cryptic spider (Pearson’s *r*_13_ = -0.457, *P* = 0.086) or quinine groups (Pearson’s *r*_8_ = 0.317, *P* = 0.373).

### Consistency of Speed-Accuracy Tactics Across Situations

**FIGURE 4:**
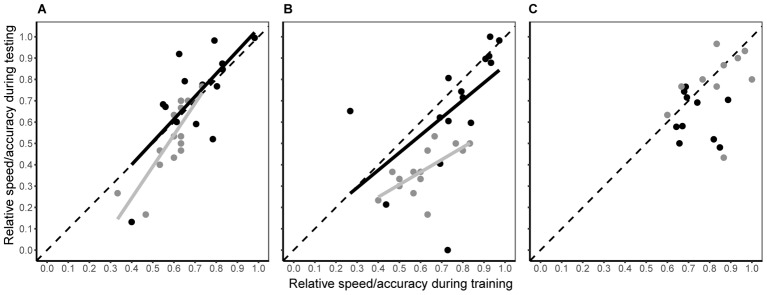
Intra-individual consistency in decision speed (black symbols) and accuracy (grey symbols) between testing and training phases for the conspicuous spider **(A)**, cryptic spider **(B)**, and quinine **(C)** groups. Fitted lines represent significant predicted values from linear regression analyses. *R*^2^ and associated *P*-values are shown in **Table 1**. To aid interpretation, the dashed grey line represents a hypothetical 1:1 relationship between testing and training phases – deviations from this show a non-uniform change in speed or accuracy along the speed and accuracy continuum.

When bees moved from a single visual discrimination task to simultaneous colour discrimination and predator avoidance (testing phase), the individual consistency in both decision speed and accuracy differed among experimental groups (**Figure 4** and **Table 1**). In the conspicuous spider group, both decision speed and accuracy during testing were dependent upon speed and accuracy during training (**Figure 4A** and **Table 1**), i.e., the rank position of individual bees remained consistent. Although there was a small (2% increase; Hedge’s g estimate = -0.131, 95% CI = -0.879 to 0.618), but significant, increase in decision speed within the group (**Table 1**), this change was consistent for all individuals (linear regression: β = 1.065 ± 0.292; contrast against a slope of 1: *F*_1_ = 0.05, *P* = 0.828). Individual accuracy within the group also fell slightly during testing (6% decrease; **Table 1**; Hedge’s g estimate = 0.601, 95% CI = -0.163 to 1.366) and this change was consistent within the group (linear regression: β = 1.481 ± 0.275; contrast against a slope of 1: *F*_1_ = 3.06, *P* = 0.104). In the cryptic spider group, the rank position of individual bees also remained constant for both speed and accuracy (**Figure 4B** and **Table 1**). Individual decision speed did not change significantly between phases (**Table 1**), but decision accuracy was strongly (by 23.6%) reduced (Hedge’s g estimate = 2.421, 95% CI = 1.136 to 3.405) during the testing phase. Furthermore, the reduction in accuracy was greater for the bees which were most accurate in training (**Figure 4B**; linear regression: β = 0.584 ± 0.176; contrast against a slope of 1: *F*_1_ = 5.62, *P* = 0.034). In contrast to the conspicuous spider and cryptic spider groups, there was no consistency in rank position, or changes in speed or accuracy between training and testing for bees in the quinine group (**Table 1**).

**Table 1:** Summary of changes in decision speed and accuracy between training and testing phases for each experimental group.

	Relative decision speed		Decision accuracy	
	Linear regression	Paired *t*-test	Linear regression	Paired *t*-test
Conspicuous spider	*R*^2^ = 0.505	*t* = 0.520	*R*^2^ = 0.691	*t* = -2.394
	*F*_13_ = 13.24	*df* = 14	*F*_13_ = 29.03	*df* = 14
	***P* = 0.003**	***P* = 0.021**	***P* < 0.001**	***P* = 0.031**
Cryptic spider	*R*^2^ = 0.311	*t* = -1.472	*R*^2^ = 0.460	*t* = -9.636
	*F*_13_ = 5.86	*df* = 14	*F*_13_ = 11.07	*df* = 14
	***P* = 0.031**	*P* = 0.163	***P* = 0.005**	***P* < 0.001**
Quinine	*R*^2^ = 0.024	*t* = -2.227	*R*^2^ = 0.120	*t* = -0.899
	*F*_8_ = 0.193	*df* = 9	*F*_8_ = 1.095	*df* = 9
	*P* = 0.672	*P* = 0.053	*P* = 0.326	*P* = 0.392


*Significant results (*P* ≤ 0.05) are shown in bold.*

### Optimal Tactic Under Different Levels of Predation Risk

**FIGURE 5:**
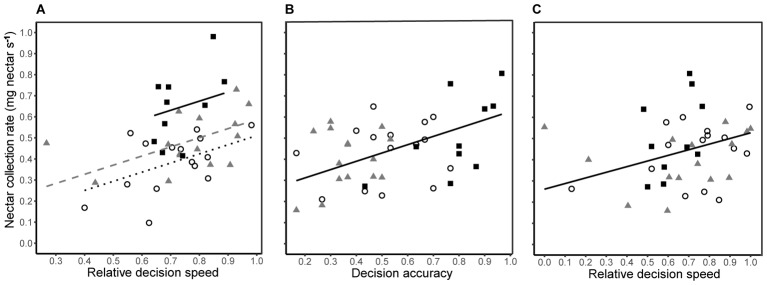
Nectar foraging efficiency was related to decision speed during the final 30 choices of training, i.e., no predation risk **(A)**, and a combination of decision accuracy **(B)** and decision speed **(C)** during the final 30 choices of testing, i.e., under predation threat. In all panels, nectar collection rates of bees from each group are shown by different symbols (conspicuous spider = open circles; cryptic spider = grey triangles; quinine = solid black squares). **(A)** The lines represent predicted values from a significant linear regression model (*R*^2^ = 0.481, *F*_1,36_ = 11.13, *P* < 0.001) with a common slope but different intercepts for each group (conspicuous spider = black dotted line; cryptic spider = dashed grey line; quinine = solid black line. **(B)** Individual nectar collection rates during the testing phase are shown against decision accuracy and the black solid line represents predicted values from the linear regression (nectar collection rate ∼decision accuracy + decision speed; *R*^2^ = 0.185, *F*_1,13_ = 8.63, *P* = 0.006 ) with values for speed set at the group mean value. **(C)** Nectar collection rates are plotted against decision speed and the black solid line represents predicted values from the same linear regression as in **(B)** but with values for accuracy set at the group mean value. In all cases, the regression lines are not extrapolated beyond observed values.

The time taken to visit 200 flowers (natural log) was negatively correlated with relative decision speed during both training (Pearson’s *r*_38_ = -0.570, *P* < 0.001) and testing (Pearson’s *r*_38_ = -0.483, *P* = 0.002) phases. The nectar collection rate (foraging efficiency) during training was dependent upon decision speed (**Figure 5A**; linear regression: *R*^2^ = 0.481, *F*_1,36_ = 11.06, *P* < 0.001) and differed between treatment groups (**Figure 5A**; linear model: *F*_2,36_ = 9.64, *P* = 0.003). A bee with 10% higher relative decision speed had a 20% greater nectar collection rate (linear model: β = 0.434 ± 0.140). In contrast, there was no difference in nectar collection rates between groups during testing (linear model: *F*_2,35_ = 1.08, *P* = 0.351), and nectar collection rates were dependent upon a linear combination of decision accuracy and decision speed (linear regression: *R*^2^= 0.318, *F*_2,37_ = 8.63, *P* < 0.001). Decision accuracy explained twice as much variation (25%; β = 0.393 ± 0.102) in nectar collection rate as decision speed (12%; β = 0.266 ± 0.099).

**FIGURE 6:**
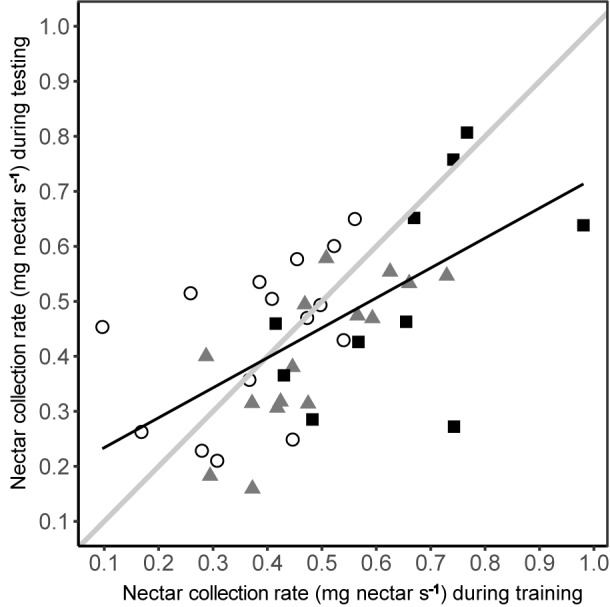
Nectar collection rate under predation risk was related to the nectar collection rate during training in the absence of predation risk. Nectar collection rates of bees from each group are shown by different symbols (conspicuous spider = open circles; cryptic spider = grey triangles; quinine = solid black squares). The solid black line represents the predicted values from a linear regression of nectar collection rate during testing against nectar collection rate during training. To aid interpretation, the solid grey line represents a hypothetical 1:1 relationship between testing and training phases – deviations from this show a non-uniform change in nectar foraging rate, i.e., the better nectar foragers during training become proportionally worse during testing than poorer nectar foragers.

The nectar collection rate under predation risk was dependent upon nectar collection rate during training (linear model: *R*^2^= 0.337, *F*_1,38_ = 19.27, *P* < 0.001), irrespective of experimental group (**Figure 6**). The relationship was positive, with the ranking of individual bees being consistent between phases, although the nectar collection rate during testing did not consistently match that during training (**Figure 6**). While most bees had lower nectar collection rates during testing (linear regression: β = 0.513 ± 0.117; contrast against a slope of 1: *F*_1_ = 17.35, *P* < 0.001) a few bees, especially those in the conspicuous spider group, had higher nectar collection rates during testing.

## Discussion

We demonstrated that while the inter-individual expression of speed-accuracy tactics remained consistent under increased predation threat, they were flexible across changing situations. However, the consistency of the speed-accuracy tactics, and degree of flexibility, were dependent upon predator detectability and the costs of making errors in the colour discrimination task. When predators were easily detected, the relationship between colour discrimination speed and accuracy was consistent when predation threat increased. In contrast, when predator detection was difficult, the relationship between decision speed and accuracy broke down, especially when errors in the floral colour discrimination task were punished with bitter quinine. Although, caution is required interpreting the results of the quinine group due to a smaller sample size and low variation in speed and accuracy among individuals. While we did detect limited flexibility in the speed-accuracy tactics employed by individual bees, there was no evidence to support our hypothesis that bees employ an optimal (in terms of nectar collection rate) speed-accuracy tactic to match the level of predation risk and detectability of the predators. Therefore, we suggest that while perceptual and cognitive constraints in bumblebees may limit the flexibility of speed-accuracy tactics employed by individual bees, a diversity of individually consistent behavioural traits at the colony level may be advantageous in environments with fluctuating predation risk (Muller and Chittka, 2008).

### Consistency of Speed-Accuracy Trade-Off Tactics Across Situations

Even though the ranking of individual speed-accuracy tactics remained consistent with increased predation threat, there were changes in the magnitude of both speed and accuracy that differed among groups. The observed consistency in ranking of bees matches a previous study where the cost of making errors in colour discrimination was increased by use of gustatory punishment (Chittka et al., 2003). In their study, Chittka et al. (2003) found that bees shifted toward the slower-accurate end of the speed-accuracy continuum when errors were punished. In contrast, in our study (which unlike the earlier study, involved predation risk), we found that bees became less accurate, with little change in decision speed, when they had to solve a difficult colour discrimination task under predation threat. The change in accuracy was only minimal when spiders were conspicuous and was coupled with a very small increase in decision speed. This most likely reflects continued improvement of predator detection after training. In the quinine treatment, which was similar to that used by Chittka et al. (2003), we found no change in either speed or accuracy under predation risk, although interindividual variation was low during both phases. The results from this group did, however, support the findings from Chittka et al. (2003) which showed increased accuracy with the addition of gustatory punishment. These observations lead us to ask two important questions. First, why did predator detectability and the cost of errors affect the relationship between decision speed and accuracy under predation threat? Second, why did bees become less accurate at choosing the most rewarding flowers when under predation threat?

To answer these questions it is worth considering how bees process the visual information relating to predation risk and food rewards. During training, bees needed to discriminate between similar coloured flowers to maximise their foraging returns. Thus, when a bee perceived a flower it should have processed the visual appearance of the flower and matched it with the level of reward it received at similar flowers (Dyer and Chittka, 2004; Dyer et al., 2011). However, when a bee was exposed to the same flowers, but under predation risk, it needed to assess both the risk associated with feeding from a particular flower as well as the difference in reward it may receive (Ings and Chittka, 2008, 2009; Wang et al., 2013; Nityananda and Chittka, 2015). Bees could either (1) scan for predators then process the floral colour, (2) process the floral colour and then scan for predators, (3) simultaneously process floral colour and scan for predators, (4) just scan for predators and visit any safe flower, or, (5) avoid the risky flower type once they have made the association between colour and predation risk.

While bumblebees are believed to use restricted parallel-like search (Morawetz and Spaethe, 2012) they still process scenes sequentially using active vision (Nityananda et al., 2014). Furthermore, although discrimination of contrasting colours requires shorter integration times than highly similar colours (Nityananda et al., 2014), bumblebees use a colour independent search image for spiders (Ings et al., 2012), i.e., complex shape recognition, that would also require longer integration times (Nityananda et al., 2014). It is therefore unlikely that they simultaneously process similar floral colours and scan for predators, although they can solve both discrimination tasks concurrently if strongly incentivised (Wang et al., 2013). Bees might avoid the risky flower type when spiders were cryptic and all flowers were rewarding (Ings and Chittka, 2009), but not when the alternative flower type is distasteful. Once bees make the association between predation risk and the highly rewarding flower type, they would only need to discriminate floral colour to avoid predation and reduce overall decision time. While bees exposed to cryptic spiders when both flowers were rewarding did indeed reduce their accuracy, they still visited too many safe high rewarding flowers to reflect avoidance of the risky flower type. It is therefore more likely that reduced accuracy reflects avoidance of spiders first followed by less accurate decisions in the colour discrimination task. This interpretation is supported by the fact that individual bees did not spend more time overall making decisions (decision speed did not change), even though increased inspection times are required to detect cryptic spiders (Ings et al., 2012). Thus, to be able to maintain overall decision times, bees will have had less time to choose between high and low reward flowers due to time lost searching for cryptic spiders. This tactic, i.e., avoiding spiders as the top priority, and then foraging from any safe flower irrespective of reward, should yield greater rewards than a tactic avoiding all high reward flowers. Further evidence for bees using this tactic is given by the fact that a similar pattern was seen for the group of bees exposed to conspicuous spiders, although the accuracy only dropped slightly, reflecting the shorter amount of additional time needed to detect the conspicuous compared to cryptic spiders (Ings et al., 2012).

Due to the importance of inspection time in calculating SAT, it is worth considering how its measurement may have influenced the observations. It could be argued that our measurement of inspection time, i.e., duration within a 7 cm × 9 cm × 9 cm zone in front of the flowers, may not capture the full decision process, thus leading to overestimated speed. While this is possible, inspection time and overall time between choices was highly correlated. Recent work has also shown that bumblebees may use active vision to distinguish complex patterns (spider shape) and similar colours, and that this requires side-to-side scanning of the scene (Nityananda et al., 2014). Such scanning behaviour has already been demonstrated to occur within the decision zone used in our experimental paradigm, especially when spiders are cryptic (Ings et al., 2012). We are therefore confident that our measure of inspection time does indeed accurately represent decision speed.

### Optimal Tactic Under Different Levels of Predation Risk

Our results showed that overall foraging duration was related to decision speed, with faster foraging bouts corresponding with faster decisions (**Figure 5**). The importance of decision speed was further borne out by the observation that foraging efficiency (nectar collection rate) in predator free environments was positively related to decision speed, but not accuracy (**Figure 5A**). Even though the two similar coloured flower types yielded very different rewards, visiting more flowers per unit time yielded a greater foraging efficiency. In contrast, decision accuracy was more important in determining foraging efficiency under predation threat, although decision speed still had some influence on foraging efficiency (**Figures 5B,C**). These results partially support the theoretical study of Burns (2005), which, using data from Chittka et al. (2003), predicted that fast, inaccurate decisions would be optimal in predator free environments, but slow, accurate decisions would be optimal under predation threat. Furthermore, a slow, accurate tactic should be favoured under predation threat because the proportion of “safe” rewarding flowers was low (Burns and Dyer, 2008) compared to when there was no predation risk. An alternative explanation could relate to the importance of avoiding being killed by a predator, such that the optimal foraging tactic under predation threat is to avoid predators irrespective of the cost to foraging efficiency. Indeed bees do maintain high levels of predator avoidance accuracy, despite the costs of detecting predators (Ings and Chittka, 2008, 2009).

An important point to consider is that although fast decisions were better in the predator free environment, and accurate decisions were better under predation threat, individual bees did not shift their tactic sufficiently to match the level of predation risk. This is borne out by the observation that nectar collection rates under predation risk were lower than those during training, even though they were strongly related. This reflects the fact that shifting behaviour to match the optimal tactic for changing situations can be costly when acquisition of information is difficult (DeWitt et al., 1998), or when the environment changes rapidly (predation risk in our case). In such situations, colonies with a diversity of individual speed-accuracy tactics (Burns and Dyer, 2008; Pruitt and Riechert, 2011), analogous to bet-hedging genotypes (Seger, 1987), could, on average, perform better in environments with fluctuating predation risk.

## Conclusion

Our study has shown that, bumblebees, which have evolved in fluctuating environments, show a degree of flexibility of speed-accuracy tactics in response to changing predation threat. However, no individual speed-accuracy tactic resulted in optimal foraging efficiency under different levels of predation threat. We suggest that this reflects perceptual and cognitive constraints that limit the flexibility of tactics expressed by individual bees. One possibility is that, as for many other traits in social species (reviewed in Jandt et al., 2014), including behavioural traits (Muller and Chittka, 2008), the diversity of speed-accuracy tactics at the colony level may be more important than individual tactics that are optimal under set circumstances. However, further work is required to test this possibility.

## Data Availability Statement

The raw data supporting the conclusions of this manuscript will be made available by the authors, without undue reservation, to any qualified researcher.

## Ethics Statement

Only commercially reared bumblebees were used in this study and our protocol conformed to the regulatory requirements for animal experimentation in the United Kingdom.

## Author Contributions

TI and LC designed the predator avoidance paradigm. M-YW and LC designed the experiments with input from TI. Experiments were carried out by M-YW and statistical analyses were conducted by M-YW and TI with input from LC. MY-W and TI prepared figures and wrote the manuscript with contributions from LC.

## Conflict of Interest Statement

The authors declare that the research was conducted in the absence of any commercial or financial relationships that could be construed as a potential conflict of interest.
